# Specific detection of foot-and-mouth disease serotype Asia 1 virus by carboxyl-magnetic beads conjugated with single-domain antibody

**DOI:** 10.1186/s12896-015-0201-5

**Published:** 2015-09-15

**Authors:** Shunli Yang, Shuanghui Yin, Youjun Shang, Di Wang, Weimin Ma, Jijun He, Jianhong Guo, Jianping Cai, Xiangtao Liu

**Affiliations:** State Key Laboratory of Veterinary Etiological Biology, National Foot and Mouth Diseases Reference Laboratory, Lanzhou Veterinary Research Institute, Chinese Academy of Agricultural Sciences, Lanzhou, Gansu 730046 China

## Abstract

**Background:**

Immunomagnetic nanobead (IMNB) labeled with specific antibody, has been demonstrated to be useful for the capturing and detection of viruses.

**Results:**

In this study, we developed an imunomagnetic bead based on carboxyl-magnetic beads (MNB) labeled with a single-domain antibody (sdAb) for capturing foot-and-mouth disease (FMD) Asia 1 virus. After magnetic separation, complexes of MNB-sdAb-virus were detected with either a sandwich ELISA or QDs-C5 probe under a fluorescence microscope, and the complexes were used as templates for extraction of total RNA for amplification of the VP1 or 3D gene fragments using RT-PCR and real-time RT-PCR. The Asia 1 VLPs were efficiently captured through IMNB with a high binding rate of 5.09 μg of antigen/μl of bead suspension. Moreover, this method has been successfully used to capture Asia 1 antigen in synthetic samples.

**Conclusion:**

Ultimately, a specific and highly sensitive capture FMDV Asia 1 tool has been established that has the potential to enhance the sensitivity and reliability when diagnosing FMDV Asia 1.

**Electronic supplementary material:**

The online version of this article (doi:10.1186/s12896-015-0201-5) contains supplementary material, which is available to authorized users.

## Background

Foot-and-mouth disease (FMD) is one of most contagious diseases of cloven-hoofed animals including cattle, pigs, sheep, buffalo, and approximately 70 wildlife species. The disease has been identified worldwide where livestock are raised. In the last 20 years, there have been massive outbreaks of FMD in countries formerly free of the disease, such as in Taiwan in 1997 [1], and in the United Kingdom in 2001 [2]. Every outbreak of disease causes enormous economic loss and significant increases in public awareness. The causative agent is FMD virus (FMDV), which consists of a single stranded positive-sense RNA genome, encoding a viral polyprotein, and is a member of the genus Aphthovirus within the family Picornavirvidae. FMDV exists in seven different serologically distinct, serotypes: O, A, C, southern African Territories 1, 2 and 3 (SAT1-3), and Asia 1. Asia 1 virus initially occurred in 1954 in Okara, Punjab, Paskistan in Asia [3, 4]. FMD Asia 1 virus is its own unique serotype, and outbreaks due to Asia 1 have been reported sporadically in the past few decades. Analyses of Asia 1 indicate that some strains have been spread across large distances between countries in Asia within a short time [5–7].

Conventional technologies, such as the virus neutralization test (VNT) [8], antigen enzyme linked immunosorbent assays (ELISAs) [9–11] and reverse transcription polymerase chain reaction (RT-PCR) [12, 13] have markedly improved the detection of FMDV antigen. However, there still exists a general need for sensitive, reliable, and fast specific viral pathogen diagnosis, isolation and identification of the different serotypes of the viruses involved in FMD outbreaks. This is crucial to the prevention and control the spread of FMD, and for minimizing the serious economic consequences that arise from an outbreak at the beginning of the pandemic. In our previous work, we screened and defined a panel of single-domain antibodies (sdAbs) against FMD Asia 1 virus, and constructed the specific probe based on conjugation of sdAb with CdSe/ZnS Quantum Dots (QDs) which were used to trace and image the subcellular location of Asia 1 virus in BHK-21 cells [14]. Herein, we extend the application of sdAb in FMDV diagnostic research. Specific Asia 1 virus sdAbs were conjugated onto the surface of carboxyl superparamagnetic beads (MNB) to form immunomagnetic beads (IMNBs) to enable them to effectively capture Asia 1 virus, forming the complexes of beads-sdAb-virus which were easy to specifically and rapidly detect using ELISA and RT-PCR. The bead- sdAb-virus complex was also labeled with QDs to measure the stable fluorescence when binding the target virus. These advantages make this a novel assay that can be used for the timely detection of FMD Asia 1 virus with high sensitivity.

## Results and discussion

A critical step is rapid and accurate detection, identification, and isolation of a target pathogen from clinical samples when there is an outbreak of disease. Magnetic beads are a powerful tool with unique advantages, such as controlled surfaces, flexible functionalization, convenience to be manipulated due to their magnetic property, and large surface-to-volume ratios where they can be widely applied to the development of analysis methods. Superparamagnetic beads conjugated with antigens and antibodies are commonly utilized in immunoassays for the enhancement of pathogen detection and separation due to their efficient enrichment and separation capability [15–18].

In this study, we developed a convenient MNB assay based on a sdAb used to specifically capture, recognize, and enrich detection of FMD Asia 1 virus (V. 1). To confirm successful conjugation of sdAb onto the surface of the functionalized MNBs capable of binding FMD Asia 1 virus, the polyclonal serum against Asia 1 antigen can be used form a sandwich ELISA with sdAb coated on the surface of MNBs in the presence of Asia 1 antigen, with the appearance of a HRP signal to indicate the successful conjugation of sdAb with MNB forming the functionalized IMNBs. The reliability and specificity of the method were further carried out by RT-PCR. This demonstrated that the complexes of virus homogeneously mixed with MNBs as all had corresponding VP1 gene fragments, which suggests that the influence of MNBs was insignificant on the extraction of total viral RNA, and was amplified template in RT-PCR reaction. The size of the 460 bp VP1 fragment of Asia 1 virus was observed in the positive samples, while the amount of PCR products of complexes of beads-virus was higher than only the culture samples. The tested samples included FMDV serotype Asia 1, A, O and were synthetic samples detected by RT-PCR after IMNB capture. Results were shown in Table 1. The IMNB has the ability to specifically bind FMDV Asia 1, moreover the type A and O samples were all negative in this study. The results suggests that IMNB may bind specifically to FMDV Asia 1, and well enhance sensitivity when diagnosing FMDV Asia 1 using RT-PCR.

**Fig. 1:**
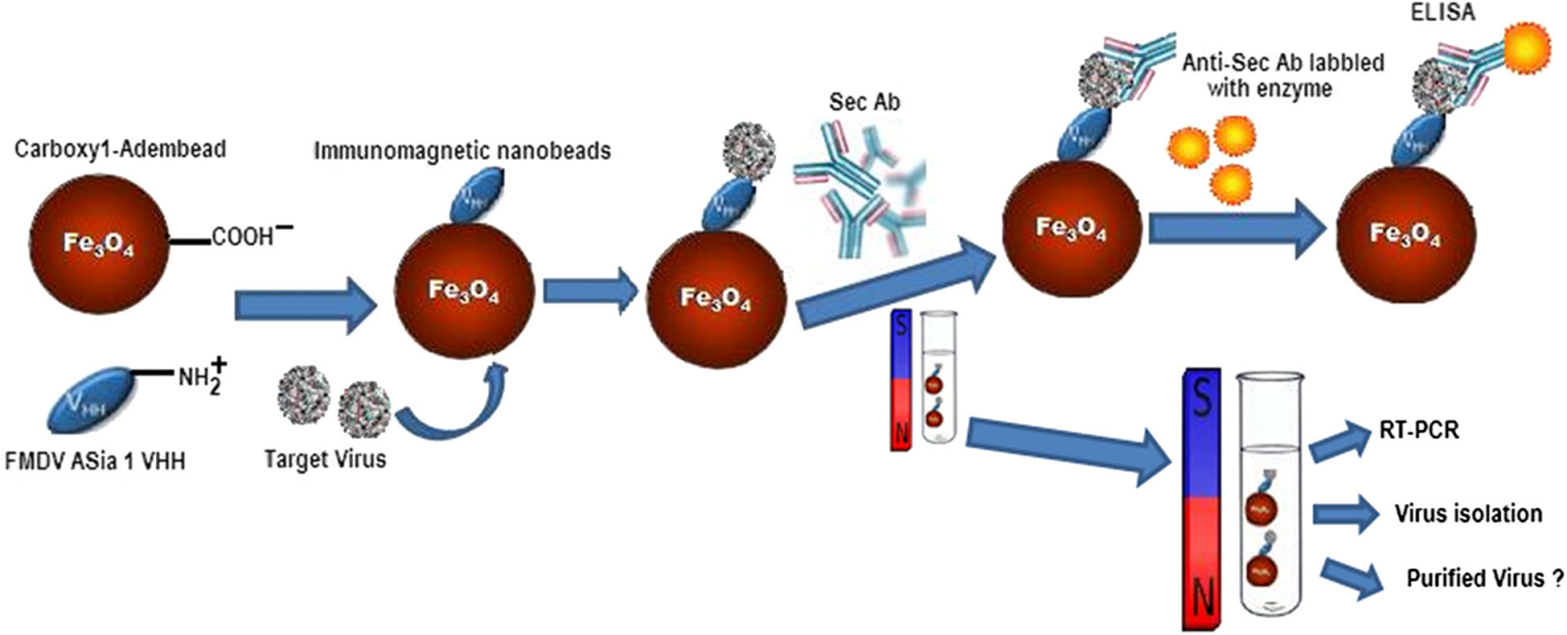
Principle for FMD Asia 1 virus detection using sdAb based on immunomagnetic separation. Carboxyl-adembead are functionalized with anti- FMDV Asia 1 sdAb forming the immunomagnetic beads, and used to capture FMDV Asia 1 in solution. The complexes of beads-sdAb-virus are easily separated from solution using an ordinary magnet. The isolated complexes may be detected by the antigen in both an ELISA and RT-PCR, and can also be utilized in virus isolation and for purifying virus

**Table 1:** Detection of the FMDV samples using IMNB

Background of Samples	FMDV Serotypes	Asia 1 Results
YNBS/58	Asia 1	P*
HNK/CHA/05	Asia 1	P
HeB/China/1/2005	Asia 1	P
HN/2006	Asia 1	P
A/GS/LX/62	Asia 1	P
synthetic sample 1	Asia 1/O	P
synthetic sample 2	Asia 1/A	P
synthetic sample 3	A/O	N*

P* and N* represent Asia 1 positive and Negative, respectively

**Fig. 2:**
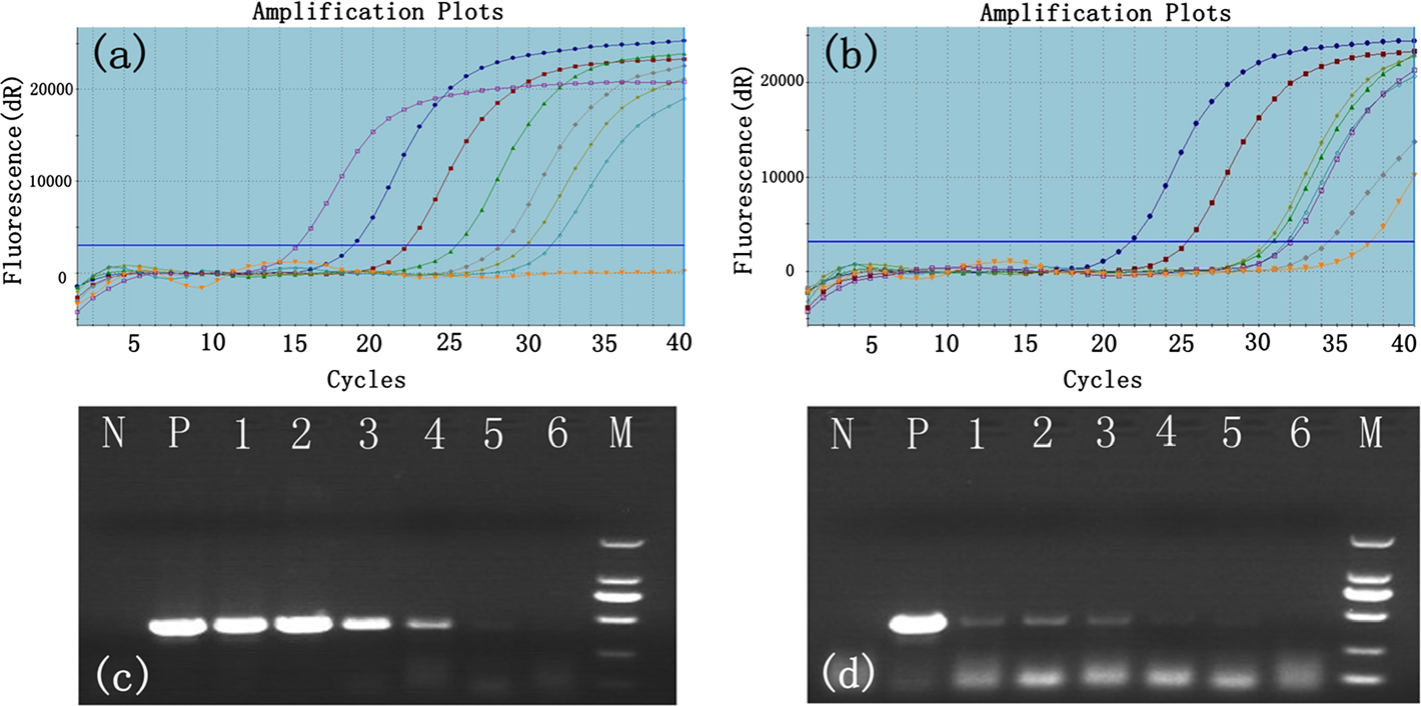
Serial dilution of primary FMD Asia 1 virus RNA in vitro was amplified to 3D fragment using real-time PCR and general RT-PCR amplifying VP1 fragment based on immunomagnetic to conjugate with sdAb. **a** FMD Asia 1 virus mixed with IMNBs at the concentration of 10^−1^ to 10^−6^ TCID_50_/mL; **b** FMD Asia 1 virus was diluted at the concentration of 10^−1^ to 10^−6^ TCID_50_/mL (**c**) lane N, negative control using sterile PBS as template; lane P, positive control of primary FMD Asia 1 virus (10^0^ TCID_50_/mL) reacted with MNB; lanes 1–6, Asia 1 virus mixed with MNB at the concentration of 10^−1^ to 10^−6^ TCID_50_/mL, respectively; lane M, DL2000 DNA marker 2000, 1000, 750, 500, 250, 100 bp; (**d**) lane N, negative control using sterile PBS as template; lane P, positive control of primary FMD Asia 1 virus (10^0^ TCID_50_/mL); lanes 1–6, FMD Asia 1 virus at the concentration of 10^−1^ to 10^−6^ TCID_50_/mL, respectively; lane M, DL2000

The conventional RT-PCR and real-time RT-PCR have been widely used to specifically detect FMDV [19, 20]. The purpose of this assay was to estimate the binding target virus efficiency of MNBs by real-time RT-PCR and conventional RT-PCR. In the first real-time RT-PCR assay, amplification of the 3D fragment, the TaqMan® amplification plot started in the 14.5^th^ cycle (Fig. 2a) which was as template with complexes of beads-virus, and comparison of the original virus as template with the amplification plot appeared in the 22^nd^ cycle (Fig. 2b) in the second real-time RT-PCR assay. The parallel conventional RT-PCR tests were performed to amplify the VP1 gene with the same samples. The results demonstrated that the reaction of viruses mixed with the IMNB all received VP1 fragments (460 bp) (Fig. 2c), which suggested the brightness of DNA strips of complexes were more than the original virus (Fig. 2d). However, it is envisioned that the method of capture of FMD Asia 1 virus with IMNB will be used in conjunction with real-time or conventional RT-PCR methods to increase the concentration of target antigen in samples per unit volume in diagnostic process and enhance the sensitivity and reliability for FMDV diagnosis.

To determine the sensitivity of IMNB, it was directly conjugated with the labeled horseradish peroxidase (HRP) sdAb-C6 (HRP-C6. The HRP-C6 was used as a second antibody to monitor the absorbance values in the sandwich ELISA. The absorbance values (OD_450_) reached a stable plateau after 35 min incubation with different concentrations of viruses from 10^−1^ to 10^−6^ TCID_50_/mL, and the absorbance values for the negative control remained unchanged (Additional file 1: Figure S1).

Some situations that may lead to the nonspecific adsorption of IMNBs when it alone was used to detect the target virus in mixtures, include natural phenomena such as hydrophobic and electrostatic interactions in this polydispersity synthetic system. In response to this, the pH and salt concentration of the reaction buffer were optimized to reduce the nonspecific background of MNBs, and to enhance the sensitivity. We also found that the largest sample to positive (S/P) ratio was obtained in PBS (0.1 mol/L, pH 7.4).

We found the ability to capture antigen of IMNB could be further reconfirmed by calculating the change of concentration of FMD Asia 1 virus VLP antigens after incubation. The P1-2A precursor is processed by viral protease 3C to produce the structural proteins VP0, VP1, and VP3 which then self-assemble to form icosahedral, empty capsid particles. [21]. The recombinant FMDV VLP contains three structural proteins, VP0, VP1 and VP3 *in vivo* when observed by SDS-PAGE [22]. We calculated that 1.0 μg of MNB (approximately 1.2 × 10^8^ beads) are able to bind 5.09 μg target antigen. Additionally, our capture efficiency assay demonstrated that the antigen complex could be dissociated by boiling, and FMD VLPs were lysed into VP0 and (~32 kDa) and VP1/VP3 (~24 kDa) (Additional file 2: Figure S2).

**Fig. 3:**
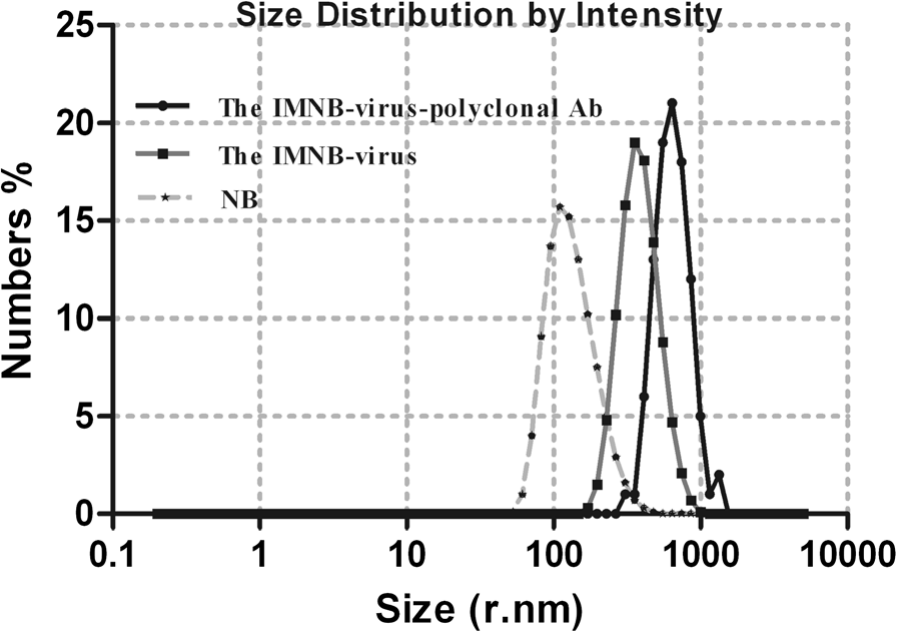
Characterzaitons of IMNBs by dynamic light scatting. The hydrated particle sizes of beads (198 nm), bead-virus complexes (356 nm), and the bead-virus-polyclonal Ab complexes (640 nm)

**Fig. 4:**
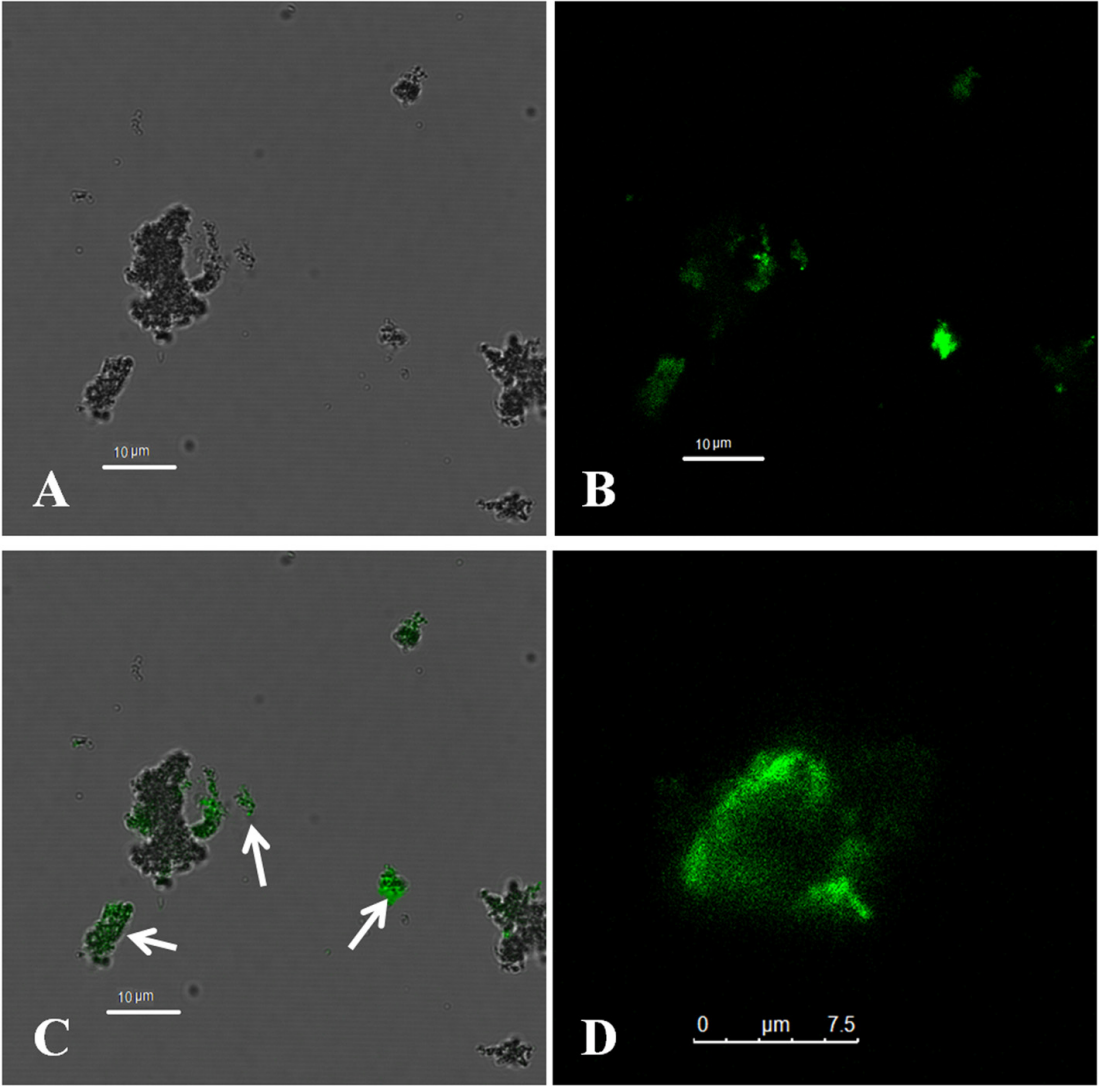
Confocal microscopic images of fluorescent, MNBs, and complexes of bead-virus. **b** The fluorescence of QDs-C5 from the labeled target virus (the green spots, on the upper left), **a** The beads in bright field image (black spots, on the upper right), **c** The merged image of complexes of beads-virus-(QDs-C5) indicating successful capture of target virus onto the MNB (on the lower left), **d** The aggregation of complexes of beads-virus-(QDs-C5) (on the lower right, as indicated by the arrows)

The capture of the viruses with the IMNBs was observed by TEM. The blackish spots are the beads, while the protuberant spots (result no shown) are characteristic of virion morphology around the beads, because the FMDV particle is too small, at approximately 25 ~ 30 nm in diameter [23] compared with the beads at about 200 nm in diameter, which leads to the virus particles not being obvious in the image. The evidence of binding virus was also suggested by the increase in the hydrated particle size of MNBs from 198 nm to 640 nm with dynamic light scatting (ZetasizerNano ZS90) (Fig. 3). Binding of the target virus to the functionalized IMNBs could be easily realized by mixing with QDs-C5 and co-localization analysis of beads, FMD Asia 1 virus and fluorescent QDs resulting in QDs-labeled virions. The functionalization of MNBs with sdAb incubated with FMD Asia 1 virus, were then reacted with QDs-C5, which produced the QDs-labeled virions. The labeled virions were evaluated by confocal fluorescence microscopy, and an overlap of functionalized MNBs between the bright field and fluorescent image (Fig. 4, green) which indicated that functionalized MNBs had successfully captured FMD Asia 1 virus was generated. The number of molecules sdAb conjugated to each MNB was estimated by the fluorescence intensity of the fluorescein-labeled VHH molecular conjugated to magnetic beads [24] and was calculated about 900–1200 protein molecular on a bead. The amount of sdAb molecule per bead showed sufficient binding antigen sites to the coupling of target virus to ensure the capture capacity of IMNBs. The combination of QDs and IMNBs offers researchers a new pathway to both separate and identify targets of interest via the use of a simple magnet, and is particularly advantageous in the detection and quantification of soluble biomarkers during which excess QD-antibody constructs must be removed for further analysis [17].

These results suggest that MNBs have a specific capture capacity of FMD Asia 1 virus, while it should be noted that future work towards the translation of this method into the field application must take into account the evaluation of a large volume of actual clinical samples.

## Conclusions

In summary, we have successfully used a specific sdAb conjugated with superparamagnetic beads forming IMNB for the rapid detection of FMD Asia 1 virus. In this method, the IMNB has the ability to efficiently capture the FMD Asia 1 virus to use in a MNB-virus-polyclonal antibody sandwich ELISA, in general RT-PCR and real-time RT-PCR. It was applied to synthetic samples of different serotypes to validate its specificity. Moreover, this method may well enhance the sensitivity and reliability when detecting FMDV Asia 1.

## Methods

FMD Asia 1 virus strain (Genbank: EF149009), sera, BHK-21 cell, real-time RT-PCR, general RT-PCR and ELISA kit used in this research were obtained from OIE/CHINA National Foot and Mouth Disease Reference Laboratory, Lanzhou Veterinary Research Institute, CAAS. The sdAbs of clone 5 (C5) and 6 (C6) anti-FMD Asia 1 virus VP1 protein were expressed in *E.coli* and purified using Ni-NTA agarose [14]. Carboxyl-adembeads super-paramagnetic beads (200 nm) were purchased from Ademtech SA (Pessac, France), 1-ethyl-3-(3-dimethylaminopropyl) carbodiimide hydrochloride (EDC) was purchased from Sigma-Aldrich (St.louis, MO, USA), QDs (505 nm, green) were obtained from Wuhan Jiayuan Quantum Dots Co.Ltd. (Wuhan, China) which were conjugated with sdAb-C5 to form QDs-sdAb clone 5 (QDs-C5).

### Fabrication of immunomagnetic Beads

According to the product instructions, the carboxyl-adembeads (33.33 μL/mg) were washed 4 times with 0.01 M phosphate buffer solution (PBS, pH 6.8), then monodispersed in 600 μL of 0.01 M PBS. The washed beads were activated in 100 mM EDC and 100 mM NHS in 400 μL of 0.01 M PBS for 30 min at 37 °C with gentle shaking. Then, they were washed with 0.01 M PBS (pH 7.4) 3 times to remove the excess reagents, and dispersed in 800 μL 0.01 M PBS. The activated beads were mixed with 150 μg of sdAb-C6 antibody in 1 mL 0.01 M PBS at room temperature for 7 h. Afterward, the immunomagnetic bead-C6 (IN-C6) was washed by 0.01 M PBS 5 times, and they were blocked with 1 mL 3 % BSA for 30 min at 37 °C. Finally, the functional beads were stored in 0.01 M PBS with 1 % BSA (m/v) and 0.05 % NaN_3_ at 4 °C for use.

### Detection of FMD Asia 1 virus using ELISA and general RT-PCR

The titer of Asia 1 virus was quantified in BHK-21 cell by 50 % tissue culture infective dose (TCID_50_). Three mg/mL(theoretically calculated value was approximately 3 × 10^8^ beads) and 1 mL inactivated virus samples of 10^6^ TCID_50_/mL Asia 1 were simultaneously mixed in 1 mL of PBS (0.1 mol/mL, pH 7.4) for a 1 h incubation at 37 °C with gentle shaking. The bead-virus complexes were separated by a magnetic scaffold to remove the suspension and washed 3 times using 0.05 % tween-20 in PBS, then resuspended in 300 μL PBS. One hundred microliter complexes were detected with a sandwich ELISA. The complexes were added to 100 μL anti-Asia 1 guinea pig serum (1:1000) for 45 min incubation at 37 °C. After washing 3 times, 100 μL of anti-guinea pig IgG-HRP at a dilution of 1:100 was added and incubated for 45 min at 37 °C. After an additional washing step, TMB substrate was mixed with complexes for 10 min at 37 °C, and the reaction was stopped with 2.0 M sulfuric acid. The optical absorbance was measured at 450 nm. Another 200 μL of complexes of bead-virus were used as the template to extract the total RNA for the general RT-PCR to amplify the VP1 fragment using the a pair of primers, Vp1F:5′-actaccaccactggcgag-3′ and Vp1R:5′- gccggttgttcactctgcg-3′. After optimizing the reacting real-time RT-PCR efficiency using the 10-fold serially diluted 10^6^ TCID_50_/mL culture virus at 10^0^, and from10^−1^ to 10^−6^ in PBS (0.1 mol/mL, p H 7.4), 200 μL and 600 μL diluent were transferred to new tubes, respectively, the former as a template to extract RNA and detect the 3D gene which was for the development of a standard curve by real-time RT-PCR, and the latter mixed with MNBs to concentrate target virus according to the process described above. The total RNA was extracted from bead-virus complexes of 600 μL sample and virus sample of 200 μL which was used as the template in the reaction of the real-time RT-PCR and general RT-PCR, respectively. The negative control was PBS buffer without target RNA in each RT-PCR assay.

### Characterizations of immunomagnetic beads

The co-localization analysis of bead-virus complexes was performed with interaction with QDs-C5 (505 nm, green). 1 mL of Asia 1 inactivated virus sample (10^6^ TCID50/mL) was mixed with 20 μL of 3 mg/mL MNBs in PBS (pH 7.4) for 45 min at 37 °C. After washing by magnetic separation, the complexes were reacted with QDs-C5 for 45 min at 37 °C with gentle shaking. After washing, the immune complexes of beads-virus-QDs-C5 were observed by a confocal fluorescence microscope (Leica, TCS SP8). The transmission electron microscopy (TEM) image of bead-virus complexes was observed by a JEM-1230 electron microscope.

All the concentrations of the beads, the hydrated particle size of the NBs, the MNB-virus complexes, and the MNB-virus-polyclonal Ab complexes, were diluted 1:100 (approximately 10^8^ beads/mL) in 0.5 mL of PBS (0.1 mol/mL, pH 7.4) according to the manufacturer’s directions (ZetasizerNano ZS90, Malvern Instruments), and were measured with dynamic light scattering.

### Capture efficiency assay

The recombinant Asia 1 VLPs at a concentration of 2.775 mg/mL in 200 μL of PBS (pH 7.4) were incubated with 50 μL of 3 mg/mL MNBs at 4 °C for overnight. After removing the supernatant under magnet separation, the beads-virus complexes were washed 3 times with PBS, added to 19 μL of PBS and 1 μL of 4× loading buffer, and processed through 12 % SDS-PAGE gel. The capture efficiency was quantified and calculated by measurement of absorbance with a NanoDrop 2000 spectrophotometer ( Thermo, Rockford, IL, USA ).

### Detection of the FMDV samples using IMNB

Tested samples or cell-culture-adapted strains of FMDV Asia 1/YNBS/58 (GenBank: AY390432), Asia 1/HNK/CHA/05 (GenBank: EF149010), Asia 1/HeB/China/1/2005 (GenBank: EF187274), Asia 1/HN/2006 (GenBank: KC412634), O/China/5/99(GenBank:HQ009509), A/GS/LX/62 (GenBank:AJ131666) and three mixtures (synthetic sample 1: Asia 1/YNBS/58 and O/China/5/99; synthetic sample 2: Asia 1/YNBS/58 and A/GS/LX/62; synthetic sample 3: O/China/5/99 and A/GS/LX/62) were used to mimic real samples, which were provided by OIE/CHINA National Foot and Mouth Disease Reference Laboratory, which were inactivated using binaryethyleneimine to evaluate the diagnostic sensitivity and specificity of IMNB.

## Additional files

Additional file 1: Figure S1. The changes of absorbance values of HRP-C6 bound to the complexes of the IMNB-virus with different incubation times by sandwich ELISA. The HRP-C6 was incubated with the complexes of IMNB-virus of 5 min, 10 min, 15 min, 20 min, 30 min and 35 min, respectively. Then the mixtures were washed for four times with PBST, add the TMB substrate was added and incubation at 37 °C for 30 min. The color reaction was measured at 450 nm. (TIFF 157 kb) Additional file 2: Figure S2. Analysis of the capture FMD Asia 1 VLPs products with IMNB by SDS-PAGE. Lane 1: VP0 and VP1/Vp3; M: molecular weight protein ladder. (TIFF 73 kb)
