# Supplementary figures and images for: Specific detection of foot-and-mouth disease serotype Asia 1 virus by carboxyl-magnetic beads conjugated with single-domain antibody
Source: BMC Biotechnol. 2015 Sep 15;15:83. doi: 10.1186/s12896-015-0201-5 (PMC4570608; doi:10.1186/s12896-015-0201-5)

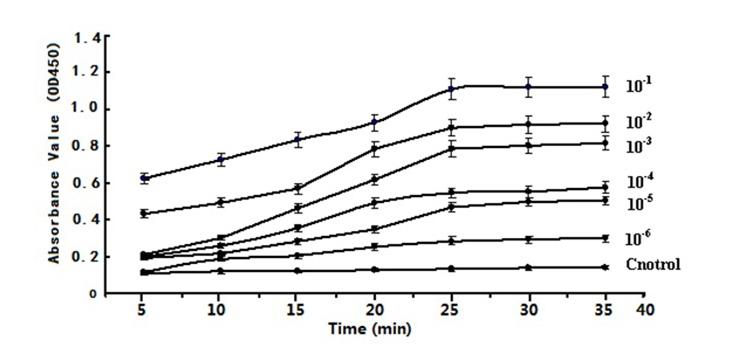

Supplement: Additional file 1: Figure S1. — The changes of absorbance values of HRP-C6 bound to the complexes of the IMNB-virus with different incubation times by sandwich ELISA. The HRP-C6 was incubated with the complexes of IMNB-virus of 5 min, 10 min, 15 min, 20 min, 30 min and 35 min, respectively. Then the mixtures were washed for four times with PBST, add the TMB substrate was added and incubation at 37 °C for 30 min. The color reaction was measured at 450 nm. (TIFF 157 kb) [file 12896_2015_201_MOESM1_ESM.tif]

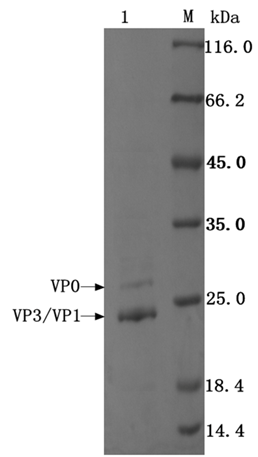

Supplement: Additional file 2: Figure S2. — Analysis of the capture FMD Asia 1 VLPs products with IMNB by SDS-PAGE. Lane 1: VP0 and VP1/Vp3; M: molecular weight protein ladder. (TIFF 73 kb) [file 12896_2015_201_MOESM2_ESM.tif]
